# Retrospective Analysis of Veterinary Pathology Laboratory Submissions to Characterise Diagnostic Patterns in South Africa

**DOI:** 10.3390/vetsci13101025

**Published:** 2026-09-28

**Authors:** Olwam Monakali, Alischa Henning

**Affiliations:** Department of Paraclinical Sciences, Faculty of Veterinary Science, University of PretoriaOld Soutpanroad (M35), Onderstepoort 0110, Gauteng, South Africa; olwam.monakali@up.ac.za

**Keywords:** veterinary pathology, veterinary diagnostic laboratory, disease surveillance, retrospective analysis, epidemiology, South Africa, business intelligence

## Abstract

Veterinary pathology laboratories play an important role in animal health, food security and disease surveillance by generating large volumes of diagnostic data. However routinely collected laboratory data is often underutilised as a source of epidemiological information. This study evaluated the potential of routinely collected veterinary pathology laboratory data to identify temporal, species-related and submitting-client location patterns in diagnostic submission in South Africa. A retrospective analysis of 3786 post-mortem cases submitted to the Onderstepoort Pathology Laboratory (OPL) between January 2018 and November 2023 was conducted using a business intelligence tool. Cases were classified as *Infectious*, *Non-infectious*, *No diagnosis* or *Complex*, and analysed according to year, season, species and submitting-client location. *Non-infectious* cases constituted the largest proportion of the diagnostic caseload (1803/3786; 48%), followed by *Infectious cases* (1532/3786; 40%). *Non-infectious* cases were particularly represented among canine and exotic and game submissions, whereas *Infectious* cases constituted a larger proportion of submissions from several production-animal groups. Seasonal variation was observed in the diagnostic caseload, with differences between pathological categories across seasons; however, these patterns were descriptive and did not establish seasonal disease risk. Gauteng accounted for 76% of submissions, followed by Northwest (8%), Limpopo (6%) and Mpumalanga (4%), reflecting the geographic distribution of submitting-client locations to a laboratory located in Gauteng rather than population-level disease occurrence. Retrospective veterinary pathology laboratory data can provide useful information on diagnostic workload and identify temporal, species and submitting-client location patterns that may contribute to veterinary disease surveillance. The findings also highlight the importance of standardised data capture, appropriate interpretation of referral-based datasets and integration of laboratory information with other surveillance streams. The 2023 caseload represents January to November only and is therefore not directly comparable with full-year totals.

## Simple Summary

Veterinary diagnostic laboratories examine animals that have died to determine why they became ill or died, generating large amounts of information that can also help identify patterns in animal diseases. However, this information is often primarily used to diagnose individual cases and remains underutilised for broader disease monitoring. This study analysed 3786 post-mortem records from the Onderstepoort Pathology Laboratory collected between 2018 and 2023 to investigate patterns according to animal species, time of year and submitting-client location. Almost half of the cases were due to non-infectious conditions, while 40% were associated with infectious diseases. The types of conditions identified differed between animal species, and differences in the number and type of cases submitted were also observed between seasons. Most submissions originated from Gauteng and neighbouring provinces, reflecting the laboratory’s location and referral patterns rather than differences in disease occurrence between provinces. The study demonstrates that information routinely collected during veterinary investigations can provide valuable insights into animal health and may contribute to complementary disease surveillance. Improving the way this information is recorded, combined with information from other laboratories and animal health programmes, could strengthen disease surveillance in South Africa and help identify important changes in animal health over time.

## 1. Introduction

The value of diagnostic data is realised when data are systematically acquired, processed, and analysed to generate actionable information [1,2]. Veterinary diagnostic laboratories generate large volumes of clinical, pathological and demographic data through routine diagnostic activities, providing an important but often underutilised source of information for disease surveillance and animal health management [1,2,3].

Disease surveillance is an integral component of veterinary health because it supports the detection and monitoring of diseases with implications for animal health, food security, trade and public health [4]. Veterinary pathology contributes to this process through the diagnosis of infectious and non-infectious conditions across multiple animal species and through the generation of information on disease patterns and causes [5,6,7]. Unlike targeted surveillance systems that are designed around specific diseases, diagnostic laboratory datasets can provide a broader overview of conditions encountered across species and geographic areas.

Laboratory diagnostic data represents an important surveillance stream because they combine clinical, pathological and demographic information [8,9]. However, these data are frequently generated primarily for individual case diagnosis rather than population-level surveillance and may therefore contain referral, sampling and information biases. Appropriate curation and interpretation are consequently essential if diagnostic datasets are to be used for broader surveillance purposes.

In South Africa, veterinary disease surveillance is supported by national and provincial veterinary services and includes both passive disease reporting and active surveillance activities for selected diseases. The South African Veterinary Strategy identified strengthening passive surveillance, improving national disease reporting and integrating laboratory information with other veterinary data priorities. Veterinary diagnostic laboratories therefore represent an important complementary source of information within the broader surveillance landscape, particularly conditions detected through routine diagnostic investigation rather than targeted surveillance programmes. The present study does not seek to replace or evaluate these established surveillance systems, but rather to assess whether routinely collected pathology submissions can provide additional descriptive information that may be useful for surveillance and laboratory monitoring.

In South Africa, veterinary pathology laboratories provide diagnostic services to a diverse animal population that includes production animals, companion animals and wildlife. The Onderstepoort Pathology Laboratory (OPL), located within the Faculty of Veterinary Science at the University of Pretoria, provides year-round anatomical pathology services to the Onderstepoort Veterinary Academic Hospital and external clients. Its diagnostic records therefore represent a potentially valuable source of information on the types of cases submitted for veterinary pathological investigation.

International studies have demonstrated that veterinary diagnostic laboratory data can be used more effectively for epidemiological purposes when records are systematically curated and analysed, including through business intelligence (BI) tools [1,2,10]. Business intelligence systems facilitate the consolidation and visualisation of complex datasets and can assist users in identifying temporal and demographic patterns and monitoring diagnostic activity.

The present study therefore aimed to retrospectively analyse OPL post-mortem records from 2018 to 2023 using a BI tool, with particular emphasis on diagnostic category, temporal variation, species representation and submitting-client location. Rather than estimating disease prevalence in the broader animal population, the study sought to demonstrate how routinely collected pathology laboratory data can be used to characterise diagnostic caseload patterns and potentially contribute to veterinary disease surveillance.

## 2. Materials and Methods

### 2.1. Study Design

A retrospective observational descriptive study was conducted using post-mortem records submitted to the OPL between January 2018 and November 2023. December 2023 records were unavailable at the time of data extraction; consequently, 2023 represents an incomplete 11-month observation period.

The study was designed as an analysis of laboratory diagnostic submissions and therefore described the OPL caseload rather than disease occurrence or prevalence within the underlying animal populations. The analytical unit was the unique post-mortem laboratory accession/submission recorded in the Universal Veterinary Information System (UVIS). Accordingly, the term ‘case’ refers to a unique post-mortem submission and should not be interpreted as necessarily representing one individual animal in every instance.

### 2.2. Setting

The laboratory received submissions from a range of clients and geographic locations, and therefore the resulting dataset represents a referral-based diagnostic caseload rather than a systematic population sample. Geographic information used in the analysis refers to the submitting-client location recorded in UVIS and was not independently verified as the location of the animal.

### 2.3. Data Collection and Cleaning

Case data were retrieved from the UVIS (Ross Group Inc., Douglasville, GA, USA), the laboratory information management system implemented in 2013 and used for accessioning, reporting, and archiving diagnostic records. Each submission was assigned a unique accession number, enabling traceability of all associated diagnostic activities. For the purposes of this study, laboratory entries linked to the same post-mortem accession were treated as components of a single analytical case.

The raw dataset comprised 24,978 laboratory entries representing all laboratory activities. Duplicate records, corrupted entries, and incomplete datasets that could not be verified were removed following cross-referencing with administrative logs. Non-post-mortem services including biopsy-only submissions, standalone toxicology and molecular testing, research samples and operational entries, were excluded. Ancillary investigations linked to a post-mortem case, including PCR, toxicology and immunohistochemistry, were consolidated under the parent post-mortem accession to prevent inflation of the number of cases. Following data cleaning and exclusion criteria, 3786 eligible unique post-mortem cases were included in the final analytical dataset. The historical extraction retained the final cleaned dataset but did not preserve sufficiently detailed stage-specific exclusion counts to reconstruct a reliable exclusion flow for every individual filtering step.

Duplicate entries were identified where the case information was a precise match to the original entry under the same accession number, with no requests for additional tests. A total of 1834 duplicate entries were removed.

### 2.4. Case Classification

Post-mortem cases were categorised as *Infectious*, *Non-infectious*, *No diagnosis*, and *Complex*. The categories were intended to be mutually exclusive and were assigned according to the final diagnostic interpretation recorded for each post-mortem submission. *Infectious* and *Non-infectious* were assigned when a primary infectious or non-infectious pathological process, respectively, could be established or was strongly supported. *No diagnosis* was assigned when the available information and investigations were insufficient to establish a definitive diagnosis. This included cases with unsuitable or insufficient material, absent or inadequate diagnostic information, or cases for which the OPL functioned as an intermediary laboratory and the final diagnostic outcome was unavailable. These cases were distinguished from *Complex* cases because no sufficiently supported diagnosis could be established. *Complex* was assigned when two or more significant interacting pathological processes were identified and supported by the available findings, but no single process could reasonably be assigned as the primary cause of the clinical presentation or death. Thus, the *Complex* category reflected the presence of multiple established or strongly supported pathological processes, whereas *No diagnosis* reflected the inability to establish a sufficiently supported diagnosis. Cases were assigned to only one category based on the final diagnostic interpretation.

Classification was performed retrospectively by the principal investigator, using the recorded diagnosis, pathologist comments and associated ancillary testing. No formal inter-reviewer assessment was performed. This limitation has been acknowledged in the manuscript.

### 2.5. Data Analysis

The records were analysed using a BI tool to evaluate patterns in the OPL diagnostic caseload according to pathological category, year, month, season, species and submitting-client location. Microsoft Excel (Microsoft Office Professional Plus, 2019, Version 16) was used as the BI platform for data interrogation, aggregation and visualisation. The cleaned OPL dataset was structured within Excel and analysed using PivotTables, PivotCharts, filters/slicers and summary calculations. An interactive dashboard was developed to facilitate exploration of the dataset according to diagnostic category, species, year, season and submitting-client location. The dashboard was used as an exploratory and descriptive analytical interface to identify patterns within the laboratory caseload; it did not constitute a real-time disease-surveillance system and was not used to estimate disease incidence, prevalence or population-level risk.

Data quality control consisted of removal of duplicate, corrupted and unverifiable records, exclusion of non-post-mortem activities, consolidation of ancillary investigations under parent accessions, and cross-referencing with administrative records. BI outputs were checked against the final cleaned dataset and annual totals reported in Table 1.

**Table 1:** Annual post-mortem caseload and distribution of pathological categories at the Onderstepoort Pathology Laboratory, 2018 to 2023. † 2023 includes January to November only; December 2023 records were unavailable at the time of data extraction.

Category	2018 (*n*)	2019 (*n*)	2020 (*n*)	2021 (*n*)	2022 (*n*)	2023 (*n*) †	Total (*n*)	Percentage (%)
**Infectious**	222	305	227	275	287	216	1532	40%
**Non-infectious**	296	349	303	301	298	256	1803	48%
**No diagnosis **	26	61	55	48	59	48	297	8%
**Complex**	6	19	39	41	27	22	154	4%
**Total**	550	734	624	665	671	542	3786	100%
**Percentage (%)**	15%	19%	16%	18%	18%	14%	100%	

Descriptive statistics were used to summarise the number and proportion of cases within each category. Annual and monthly caseloads were summarised to describe temporal variation. Seasonal patterns were examined descriptively by grouping months into summer (December–February), autumn (March–May), winter (June–August) and spring (September–November), consistent with established South African seasonal classifications. Seasonal mean values were calculated as the arithmetic mean of the case counts for each seasonal observation period in the dataset. The study period included complete seasonal observation periods from 2018 onwards, except for the final summer period, for which December 2023 records were unavailable. December 2023 was therefore not treated as a zero-count month, and no imputation or adjustment was applied to estimate the missing records. The seasonal results were therefore interpreted descriptively and were not adjusted for unequal observation periods or converted to population-based rates.

Species-specific distributions were examined by comparing the proportion of *Infectious*, *Non-infectious*, *No diagnosis* and *Complex* cases within each species group. Geographic analyses were based on the province recorded for the submitting-client location.

## 3. Results

### 3.1. Overall Caseload

A total of 3786 post-mortem cases met the inclusion criteria between 2018 and 2023. Annual caseloads for the full observation years ranged from 550 to 734 cases. The highest full-year caseload occurred in 2019 (734; 19%), followed by 2022 (671; 18%) and 2021 (665; 18%). A total of 542 cases (14%) were recorded during January to November 2023; this incomplete 11-month period should not be directly compared with the full-year total.

*Non-infectious* cases constituted the largest diagnostic category, with 1803 cases (48%), followed by *Infectious* cases with 1532 cases (40%). *No diagnosis* accounted for 297 cases (8%), while 154 cases (4%) were classified as *Complex.* See Table 1. These percentages describe the composition of the OPL post-mortem caseload and should not be interpreted as estimates of disease prevalence in the underlying animal populations.

### 3.2. Seasonal Analysis

Seasonal variation was assessed descriptively across the four pathological categories. The highest mean number of *Infectious* cases occurred in autumn (70 cases per seasonal observation period), followed by summer (67), winter (60) and spring (59). *Non-infectious* cases had the highest mean number of submissions in spring (90), followed by winter (80), autumn (71) and summer (60). *No diagnosis* cases had the highest mean number in winter (14), followed by autumn (13), spring (12) and summer (10). *Complex* cases had the highest mean in autumn (9), followed by spring (7), winter (6) and summer (4). See Figure 1. The summer estimate includes an incomplete final observation period because December 2023 records were unavailable.

These observations indicate variation in the composition of the diagnostic caseload between seasons but do not establish seasonal differences in disease risk or incidence.

**Figure 1:**
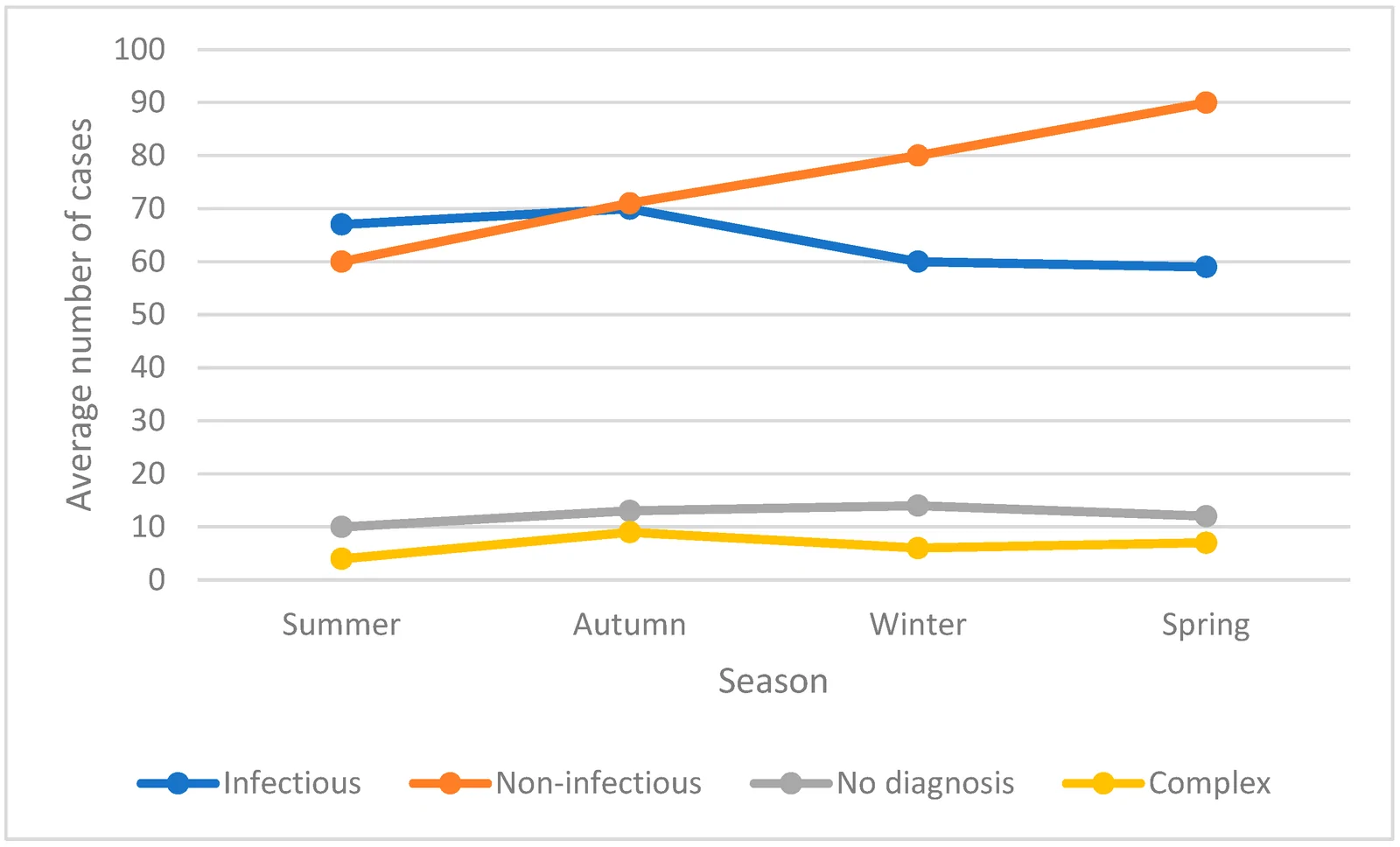
Seasonal distribution of veterinary pathology submissions by pathological category. Average number of cases represents the arithmetic mean of case counts assigned to each seasonal observation period available in the dataset; December 2023 was unavailable.

### 3.3. Species Analysis

Canine cases represented the largest species group, with 773 submissions (20%), followed by exotic and game species (725; 19%), bovine (606; 16%) and ovine (528; 14%). Caprine, equine, avian and feline cases accounted for 9%, 7%, 6% and 5%, respectively, while porcine, primate, murine, other and unknown categories collectively contributed smaller proportions of the caseload. The ‘exotic and game’ grouping represents the category recorded in the original UVIS dataset; individual taxa within this broad grouping were not separately harmonised for the present analysis. The exotic and game category included a diverse range of wildlife and captive exotic species, including antelope, rhinoceros, giraffe and other non-domestic species.

The distribution of pathological categories differed between species groups. *Non-infectious* cases represented 65% of canine submissions and 61% of equine submissions, whereas *Infectious* cases accounted for 50% of bovine, 59% of ovine, 54% of caprine and 56% of porcine submissions. Exotic and game submissions comprised 54% *Non-infectious* and 36% *Infectious* cases. See Figure 2 and Table 2. These findings demonstrate differences in the composition of the OPL diagnostic caseload between species, but they should not be interpreted as evidence that particular species have a higher population-level risk of infectious or non-infectious disease.

**Figure 2:**
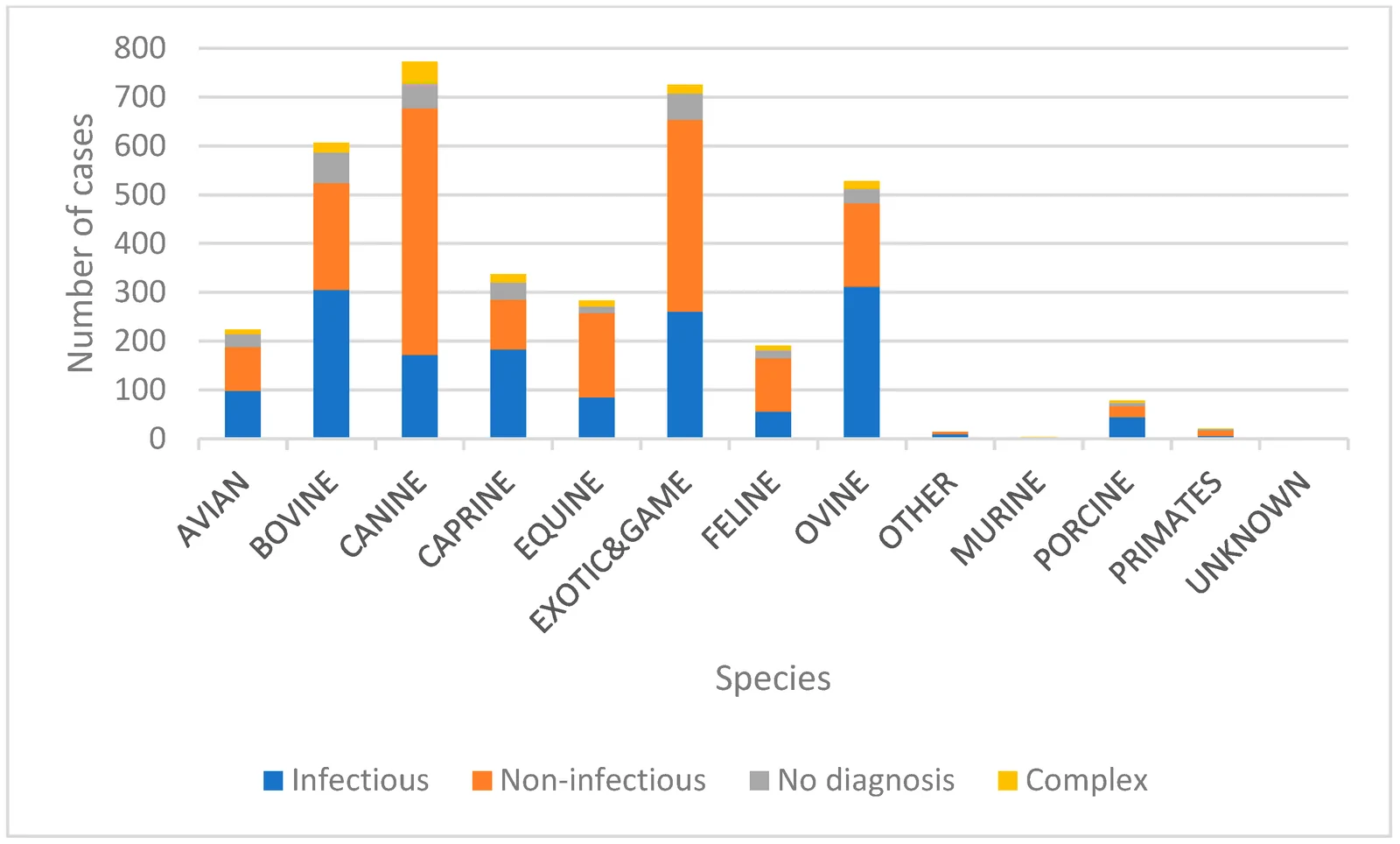
Distribution of post-mortem submissions by species and pathological category at the Onderstepoort Pathology Laboratory, 2018–2023.

**Table 2:** Distribution of post-mortem submissions by species and pathological category at the Onderstepoort Pathology Laboratory, 2018–2023.

Category	Avian (*n*)	Bovine (*n*)	Canine (*n*)	Caprine (*n*)	Equine (*n*)	Exotic&Game (*n*)	Feline (*n*)	Ovine (*n*)	Other (*n*)	Murine (*n*)	Porcine (*n*)	Primates (*n*)	Unknown (*n*)	Total (*n*)
Infectious	98	305	172	183	85	261	56	312	9	0	44	6	1	1532
Non-infectious	90	219	505	102	173	393	109	171	5	1	23	11	1	1803
No diagnosis	26	63	50	35	13	53	16	29	0	2	7	3	0	297
Complex	10	19	46	17	12	18	10	16	0	1	4	1	0	154
Overall total	224	606	773	337	283	725	191	528	14	4	78	21	2	3786
Percentages (%)	6%	16%	20%	9%	7%	19%	5%	14%	0%	0%	2%	1%	0%	100%

### 3.4. Geographic Results

Gauteng accounted for the largest proportion of submissions, with 2875 cases (76%), followed by Northwest (306; 8%), Limpopo (241; 6%) and Mpumalanga (147; 4%). The Free State contributed 76 cases (2%), while the Eastern Cape, Western Cape and Northern Cape each contributed approximately 1%. KwaZulu-Natal accounted for 15 cases. See Figure 3. Overall, 99% of submissions originated from South Africa, with a small number originating from Australia, Namibia and Swaziland. Forty-seven cases had incomplete submitting-client location information. The predominance of submissions from Gauteng and neighbouring provinces reflects the referral distribution of the OPL and its geographic location and should not be interpreted as evidence of a greater disease burden in these provinces.

**Figure 3:**
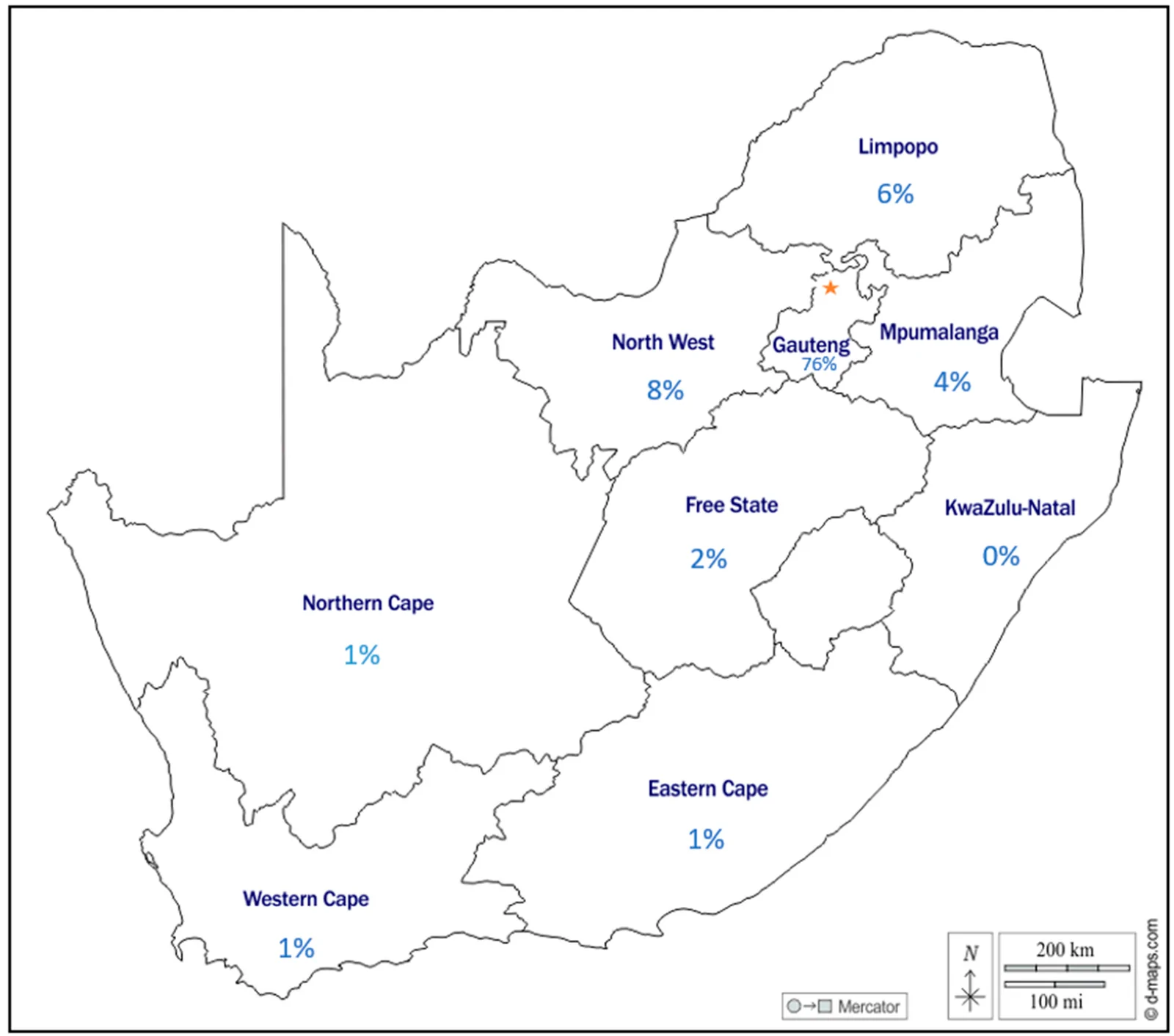
Geographic distribution of percentage (%) of post-mortem submissions to the Onderstepoort Pathology Laboratory (exact location depicted by star) by province, 2018–2023.

## 4. Discussion

The analysis of 3786 post-mortem records demonstrated that routinely collected OPL data can be used to characterise the composition of a veterinary pathology diagnostic caseload across time, species and submitting-client location. The principal value of this data lies in describing diagnostic activity and identifying patterns that may warrant further investigation, rather than estimating population-level disease occurrence. This distinction is fundamental when interpreting retrospective laboratory datasets because submission patterns may be influenced by species distribution, clinical decision-making, referral practices, accessibility of diagnostic services and client characteristics.

*Non-infectious* cases constituted the largest proportion of the OPL caseload (48%). This finding was largely associated with the high representation of canine and exotic and game submissions. Among canine submissions, 65% were classified as *Non-infectious*, while *Infectious* cases accounted for 22%. The predominance of *Non-infectious* cases among canine submissions is consistent with the broad range of chronic, degenerative, neoplastic and other non-infectious conditions for which post-mortem investigations may be requested in companion animals. However, the present data cannot determine whether these conditions are more prevalent in the wider canine population because an appropriate population denominator is unavailable. Differences in veterinary access, referral practices, client demographics and case selection may all influence the composition of submissions.

The relatively greater proportion of *Infectious* cases among production-animal submissions may similarly reflect circumstances under which production animals are submitted for post-mortem examination. Infectious disease investigations may be prioritised where multiple animals are affected, where economically important or reportable diseases are suspected, or where diagnostic confirmation is required. The observed differences between species groups therefore provide information about composition of the diagnostic workload but should not be interpreted as difference in population-level disease risk.

*Infectious* cases constituted 40% of the total OPL caseload and were proportionally prominent among several production-animal groups. Descriptive seasonal variation was also observed, with the highest mean number of *Infectious* submissions occurring during autumn, while *Non-infectious* submissions have their highest mean during spring. These patterns are descriptive and may reflect variation in submission activity, animal management, environmental conditions, disease occurrence or other factors that were not measured in the study. In addition, the incomplete 2023 observation period means that seasonal variation in infectious disease occurrence may be influenced by factors such as vector activity, environmental conditions and animal management, the present dataset does not permit these factors to be evaluated directly.

The observed seasonal pattern should therefore be regarded as a hypothesis-generating observation rather than evidence of a seasonal increase in disease incidence. Future analyses incorporating pathogen-specific diagnoses, monthly denominators, meteorological variables and relevant animal population data would be required to investigate potential environmental or seasonal associations.

The species distribution of the OPL caseload reflects the diverse animal population served by the laboratory. Canine, exotic and game, bovine and ovine submissions collectively represented a substantial proportion of the dataset. The differences in pathological-category distribution between species demonstrate the potential value of species-specific analysis of diagnostic laboratory data.

The use of laboratory information systems and BI approaches in veterinary diagnostics is consistent with previous work demonstrating that routinely generated diagnostic records can be curated and transformed into epidemiologically useful information [2,8,10]. Pozzato et al. (2021) demonstrated the application of BI tools within a multicentre veterinary pathology service, while Petukhova et al. (2023) showed how interactive dashboards can support monitoring animal pathogens [2,10]. Kloeze et al. (2012) further demonstrated the importance of a minimum dataset to facilitate collation and analysis of animal health laboratory information across jurisdictions [8]. The present study extends this general approach to a South African veterinary pathology referral dataset and illustrated both its potential utility and the limitations imposed by referral bias, incomplete metadata and the absence of population denominators.

Production-animal submissions were proportionally more frequently classified as *Infectious* than several companion-animal groups, whereas *Non-infectious* cases predominated among canine and equine submissions. These differences may be useful for identifying areas in which more detailed surveillance or targeted diagnostic monitoring could be considered but should not be interpreted as estimates of species-specific disease risk without appropriate population denominators.

The geographic distribution of submissions was strongly dominated by Gauteng, which accounted for 76% of the total caseload. Northwest and Limpopo were the next largest contributors. This distribution is unsurprising given the location of the OPL and its accessibility to clients in Gauteng and neighbouring provinces. The decreasing representation of more distant provinces may similarly reflect referral patterns, availability of alternative diagnostic laboratories and transport considerations rather than differences in disease burden.

The geographic component of the dataset nevertheless demonstrates the potential value of laboratory information systems for identifying where diagnostic services are being utilised and for describing the geographic reach of a veterinary pathology service. When combined with population denominators and data from other laboratories, such information could contribute to broader regional or national surveillance.

The potential surveillance value of the OPL dataset should be considered in relation to South Africa’s established national animal-disease reporting system. National animal-disease reports compile information submitted through provincial veterinary services and provide information on controlled and notifiable diseases and reported outbreaks. However, direct comparison with the present findings was not feasible because the OPL dataset comprised retrospective post-mortem submissions classified into the broad pathological categories, whereas national disease reports are structured around the reported disease and outbreak. The present study therefore cannot determine whether the observed annual, season or species-related patterns correspond to changes in national disease occurrence.

Temporal patterns in laboratory submissions should also be interpreted in the context of potential changes in diagnostic practice and laboratory utilisation during the study period. Changes in veterinary service availability, referral behaviours, diagnostic capacity, client access, operational procedures or external events may influence the number and composition of submission independently of changes in disease occurrence. The present dataset does not contain sufficient information to quantify the contribution of these factors. Consequently, changes in annual caseload should be interpreted as changes in laboratory submission activity rather than temporal changes in disease burden.

### 4.1. Strengths and Limitations

This study has several strengths. The final dataset comprised 3786 post-mortem cases derived from 24,978 laboratory records, with systematic removal of duplicate records and non-post-mortem-related laboratory activities. Ancillary tests linked to individual post-mortems were consolidated under the parent case, reducing the risk of artificially inflating case numbers. The study also incorporated multiple dimensions of the dataset, including pathological category, species, time and submitting-client location.

Several limitations should, however, be considered. First, the dataset represents passive, referral-based surveillance and therefore cannot be considered representative of the South African animal population. The probability that an animal undergoes post-mortem examination and is subsequently submitted to the OPL is influenced by clinical circumstances, owner or veterinarian decisions, accessibility, cost, transport and the availability of alternative diagnostic facilities. In addition, the analytical unit was a laboratory post-mortem accession/submission rather than a population-based animal unit; therefore, submission counts should not be interpreted as counts of individual animals without additional verification of the relationship between accessions and animals.

Second, the dataset does not provide appropriate population denominators for the species or provinces represented. Consequently, the proportions reported in this study represent the composition of the OPL diagnostic caseload and should not be interpreted as disease prevalence or incidence.

Third, incomplete clinical, demographic and geographic information was present in some records. Age and sex were not consistently available, and 47 cases had incomplete geographic information. The geographic variable used in this study represented the recorded submitting-client location and was not necessarily the verified location of the animal. December 2023 records were also unavailable at the time of data extraction.

Fourth, the classification of cases into *Infectious*, *Non-infectious*, *No diagnosis* and *Complex* categories relied on retrospective interpretation of recorded diagnoses, comments and ancillary investigations. Classification was performed retrospectively without a formal inter-reviewer agreement assessment, which introduces the possibility of classification variability. The distinction between *No diagnosis* and *Complex* was operationalised as described in the Methods sections, but cases with multiple or non-specific pathological process may remain subject to interpretive uncertainty. Finally, the seasonal analysis was descriptive and did not account for population denominators, changes in submission volume, meteorological variables or other potential confounders. The absence of December 2023 records further limits direct comparison of seasonal observation periods involving summer. The observed seasonal patterns should therefore be considered hypothesis-generating rather than evidence of causal seasonal effects.

### 4.2. Future Directions

The findings demonstrate the potential value of routinely collected veterinary pathology data for surveillance but also highlight the importance of standardised data capture and classification. International veterinary diagnostic laboratory initiatives have demonstrated the value of structured datasets and standardised disease classifications for epidemiological analysis [2,8,10].

For South African veterinary pathology laboratories, development of a standardised minimum dataset incorporating species, age, sex, submitting-client location, production system, clinical syndrome, final diagnosis and relevant laboratory investigations would improve the comparability and surveillance value of diagnostic records. In addition, future BI systems could incorporate natural language processing (NLP) and other artificial-intelligence approaches to extract structured diagnostic information from unstructured fields such as pathologist comments and free-text final diagnoses. Such approaches could reduce dependence on manual coding, improve consistency of disease terminology and facilitate automated aggregation of diagnoses for surveillance dashboards. Any AI-assisted extraction would, however, require validation against expert-curated records before being used for surveillance purposes. Integration of data from multiple veterinary laboratories would further reduce the referral bias inherent in single-laboratory datasets and could provide a more representative national picture.

Future studies could also link laboratory records with external datasets, including animal population denominators, meteorological information, vector activity and disease-control programme data. Such integration would allow the transition from descriptive analysis of laboratory submissions to more formal epidemiological assessment of disease risk and temporal or spatial associations.

## 5. Conclusions

This study demonstrates the potential value of routinely collected veterinary pathology laboratory data for describing patterns in diagnostic submissions across species, time and submitting-client location. Analysis of 3786 post-mortem cases from the OPL identified differences in the composition of *Infectious*, *Non-infectious*, *No diagnosis* and *Complex* cases across species and seasons, while also demonstrating the strong geographic influence of referral patterns on the laboratory caseload.

The findings demonstrate the potential for pathology laboratory data to serve as a complementary surveillance information stream, but they do not establish surveillance performance, disease prevalence, incidence, population-level risk or representativeness. The findings support the use of curated veterinary pathology data for descriptive laboratory surveillance and laboratory management. However, referral bias, incomplete data, the incomplete 2023 observation period and the absence of population denominators mean that the findings should not be interpreted as estimates of disease prevalence or incidence. Strengthening standardised data collection and integrating pathology data with information from other veterinary laboratories and surveillance systems could improve the contribution of veterinary pathology laboratories to national animal-health surveillance in South Africa.

## Data Availability

The raw data supporting the conclusions of this article will be made available by the authors on request.

## Abbreviations

The following abbreviations are used in this manuscript:

BI	Business Intelligence
OPL	Onderstepoort Pathology Laboratory
UVIS	Universal Veterinary Information System
